# Mechanistic Insights into the Anti-angiogenic Activity of *Trypanosoma cruzi* Protein 21 and its Potential Impact on the Onset of Chagasic Cardiomyopathy

**DOI:** 10.1038/srep44978

**Published:** 2017-03-21

**Authors:** Samuel Cota Teixeira, Daiana Silva Lopes, Sarah Natalie Cirilo Gimenes, Thaise Lara Teixeira, Marcelo Santos da Silva, Rebecca Tavares e Silva Brígido, Felipe Andrés Cordero da Luz, Aline Alves da Silva, Makswell Almeida Silva, Pilar Veras Florentino, Paula Cristina Brígido Tavares, Marlus Alves dos Santos, Veridiana de Melo Rodrigues Ávila, Marcelo José Barbosa Silva, Maria Carolina Elias, Renato Arruda Mortara, Claudio Vieira da Silva

**Affiliations:** 1 Laboratório de Tripanosomatídeos, Departamento de Imunologia, Instituto de Ciências Biomédicas, Universidade Federal de Uberlândia, MG, Brasil; 2 Laboratório de Bioquímica e Toxinas Animais, Instituto de Genética e Bioquímica, Universidade Federal de Uberlândia, MG, Brasil; 3 Center of Toxins, Immune Response and Cell Signaling (CeTICS), Instituto Butantan, São Paulo, São Paulo, Brasil; 4 Laboratório de Patologia Molecular e Biotecnologia do Centro de Referência Nacional em Dermatologia Sanitária/Hanseníase, Faculdade de Medicina, Universidade Federal de Uberlândia, MG, Brasil; 5 Laboratório de Osteoimunologia e Imunologia dos Tumores, Departamento de Imunologia, Instituto de Ciências Biomédicas, Universidade Federal de Uberlândia, MG, Brasil; 6 Departamento de Microbiologia Imunologia e Parasitologia, Escola Paulista de Medicina, Universidade Federal de São Paulo, SP, Brasil

## Abstract

Chronic chagasic cardiomyopathy (CCC) is arguably the most important form of the Chagas Disease, caused by the intracellular protozoan *Trypanosoma cruzi*; it is estimated that 10–30% of chronic patients develop this clinical manifestation. The most common and severe form of CCC can be related to ventricular abnormalities, such as heart failure, arrhythmias, heart blocks, thromboembolic events and sudden death. Therefore, in this study, we proposed to evaluate the anti-angiogenic activity of a recombinant protein from *T. cruzi* named P21 (rP21) and the potential impact of the native protein on CCC. Our data suggest that the anti-angiogenic activity of rP21 depends on the protein’s direct interaction with the CXCR4 receptor. This capacity is likely related to the modulation of the expression of actin and angiogenesis-associated genes. Thus, our results indicate that *T. cruzi* P21 is an attractive target for the development of innovative therapeutic agents against CCC.

Chagas disease (also known as American trypanosomiasis) is caused by the intracellular parasite *Trypanosoma cruzi* and affects 8–10 million people worldwide being endemic in Latin America where approximately 109 million individuals are at risk^1^. Chronic Chagasic Cardiomyopathy (CCC) is possibly the most important form of Chagas disease; it is estimated that 10–30% of chronic patients develop this clinical manifestation^2^. CCC is characterized by morbidity and early mortality in the most productive age group, which has created an extensive economic and social burden in endemic areas^3^,^4^,^5^.

The most common and severe form of CCC can be related to ventricular abnormalities such as heart failure, arrhythmias, heart blocks, thromboembolic events and sudden death. It is estimated that 20,000 deaths occur annually in endemic areas due to CCC complications. Moreover, refractory heart failure and thromboembolism are the most important causes of death^6^. These clinical manifestations have contributed to Chagas disease becoming one of the most important causes of heart disease^7^,^8^.

The exact mechanism of CCC pathogenesis is complex and remains unclear. However, several studies have shown that CCC etiology is likely the result of a number of factors that involve the *T. cruzi* strain, including parasite-dependent myocardial damage^9^, persistent parasitemia^10^,^11^,^12^, immune-mediated myocardial injury (autoimmunity)^13^ and host-related genetic factors^14^.

Our research group has recently characterized a recombinant protein from *T. cruzi* named P21 (rP21) as an attractive therapeutic target for CCC treatment. It has been shown that rP21 can recruit immune cells, induce myeloperoxidase and interleukin (IL)-4 production and decrease blood vessel formation compared to the controls *in vitro* and *in vivo*^15^. Here we aimed to investigate the anti-angiogenic activity of rP21 in order to get novel insight into the potential impact of the native protein on the onset of chagasic cardiomyopathy.

## Results and Discussion

### Anti-angiogenic activity of rP21 depends on its direct interaction with endothelial cells

**Figure 1:**
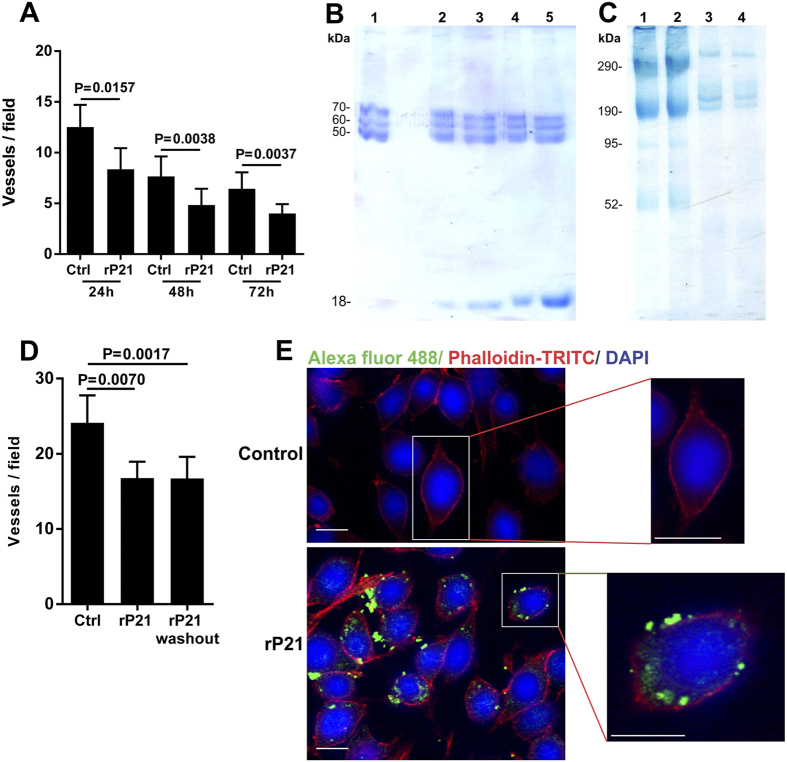
Anti-angiogenic activity of rP21 depends on its direct interaction with endothelial cells. (**A**) rP21 inhibited angiogenesis *in vitro* in a time-dependent manner at 24, 48 and 72 h. (**B**) rP21 was incubated with 50 μL of different substrates for 1 h at 37 °C. **1** Bovine fibrinogen. **2–5** Bovine fibrinogen incubated with 5, 10, 20 and 40 μg of rP21, respectively. (**C**) **1** Matrigel control (without rP21). **2** Matrigel incubated with 40 μg of rP21. **3** Fibronectin solution (without rP21). **4** Fibronectin incubated with 40 μg of rP21. rP21 was not able to promote the degradation of the ECM components. (**D**) tEnds cells pre-incubated with rP21 were washout with PBS and centrifuged. The mechanical removal of rP21 post-treatment did not interfere with its anti-angiogenic activity in cells grown on a thin layer of ECM (Matrigel). (**E**) rP21 binds to ECs and is internalized by these cells. The data are expressed as the means ± standard deviations of experiments performed in triplicate. Significant differences were determined using Student’s t-test (two-sided) and Mann-Whitney test (**A**) and one-way ANOVA and Tukey’s multiple comparisons test (**B**). Differences were considered significant when p < 0.05. kDa: kilo Daltons. rP21 = 18 kDa. AlexaFluor 488 (rP21 label). Bars: 20 μm.

Our previous studies showed that a murine endothelial cell line (tEnd) treated with different concentrations of rP21 did not exhibit altered cell viability or adhesion to a thin layer of extracellular matrix, but vessel formation was inhibited after 18 hours of incubation^15^. However, an intriguing question was raised: how does P21, a parasite-derived molecule, significantly interfere with the angiogenesis process? Therefore, in this study we aimed to gain insight into the mechanism by which rP21 inhibits angiogenesis. Thus, we verified that the anti-angiogenic activity of rP21 was time-dependent and was observed at 24, 48 and 72 h (Fig. 1A). The significant decreases in the number of vessels in the control group at 48 and 72 h were due to the intense cellular proliferation that closed the vessels.

Moreover, we evaluated the influence of rP21 upon extracellular matrix (ECM) components and observed that the protein was not able to promote degradation of bovine fibrinogen, Matrigel or fibronectin compared to the control group (untreated), regardless of the protein quantity (Fig. 1B,C). In addition, to confirm whether the anti-angiogenic activity of rP21 occurs by a direct interaction with ECM components or by interaction with endothelial cells (ECs) we incubated the tEnd cells with rP21 and the cells were then washed out or not then plated on a thin ECM (Matrigel) layer. Our results showed that in both situations rP21 was able to inhibit the angiogenesis process indicating that this protein directly interacts with ECs (Fig. 1D).

ECM regulates different cellular processes including growth, migration, differentiation, survival, homeostasis, and morphogenesis. ECM consists of a large variety of matrix macromolecules such as collagens, elastin, fibronectin (FN), laminins, glycoproteins, proteoglycans (PGs), and glycosaminoglycans (GAGs) that associate with each other to build the complex three-dimensional matrix network^16^,^17^. In this sense, Matrigel (a solubilized basement membrane-like composite from Engelbreth-Holm-Swarm sarcoma) was used as a surrogate for ECM and is mainly composed of laminin, type IV collagen, nidogen and heparan sulfate proteoglycans^18^. However, rP21 showed no detectable proteolytic activity on the matrix components (Fig. 1B,C).

Although rP21 did not interfere with EC adhesion or cellular viability^15^ and did not exhibit proteolytic activity on ECM components, this protein strongly inhibits vessel formation. This anti-angiogenic effect of rP21 could be explained by other mechanisms such as a direct interaction of rP21 with ECs. To test this hypothesis, we showed that rP21 binds to ECs and is internalized by these cells (Fig. 1E). These data suggest that the rP21-EC interaction can initiate a cascade of intracellular events that culminate in decreased vessel formation.

### The rP21-CXCR4 interaction decreases blood vessel formation and inhibits EC proliferation

**Figure 2:**
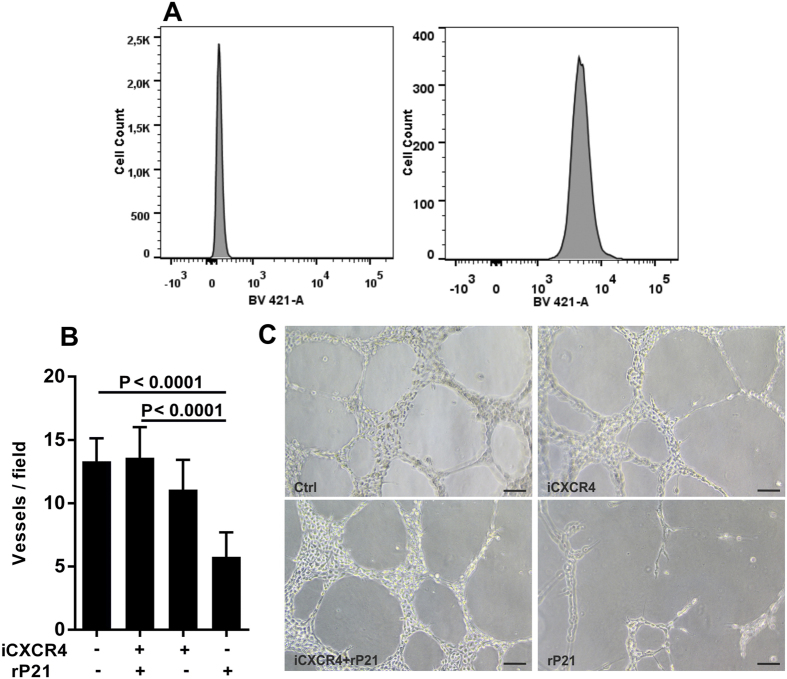
rP21-CXCR4 interaction decreases blood vessel formation. (**A**) tEnd cells showed increased CXCR4 expression on their surface. (**B**) rP21 depends on its direct binding to the CXCR4 receptor to inhibit vessel formation. (**C**) Representative phase contrast images of the organization of the tEnd cells in the Matrigel tube formation assay are shown. Data are shown as means ± SEM obtained from four independent experiments performed in triplicate. Significant differences were determined using one-way ANOVA and Tukey’s multiple comparisons test. Differences were considered significant when p < 0.05. Bars: 20 μm.

Our previous studies showed that rP21 has the capacity to bind to CXC receptor 4 (CXCR4; CD184), enhancing macrophage phagocytosis and actin polymerization^19^. Therefore, we performed additional experiments to gain insights into the rP21-CXCR4 interaction in ECs. First, we showed that CXCR4 was highly expressed on the surface of tEnd cells (endothelial cells) (Fig. 2A). Then, to assess whether the rP21-mediated inhibition of vessel formation depends on its binding to the CXCR4 receptor, tEnd cells were treated with a receptor-specific antagonist, AMD3100 (CXCR4 inhibitor). We observed that blocking CXCR4 signaling pathway and subsequent treatment with or without rP21 did not inhibit vessel formation compared to the other groups (Fig. 2B,C). Therefore, we suggest that the anti-angiogenic activity of rP21 depends on its interaction with CXCR4.

CXCL12 [stromal cell-derived factor-1 (SDF-1)] is a chemokine and primarily binds to CXCR4. CXCL12 secretion is associated with tissue damage such as heart infarction and focal ischemia to promote vessel repair. CXCR4 is expressed by T-lymphocytes, B-lymphocytes, macrophages, neutrophils, and endothelial and epithelial cells. This receptor is widely expressed by ECs in damaged tissues such as injured carotid arteries and atherosclerotic plaques during pathological processes^20^,^21^. It is known that pro-angiogenic CXCR4^+^ cells are recruited to damaged tissues along a CXCL12 gradient to promote the re-vascularization of the injured areas demonstrating the importance of the CXCL12/CXCR4 pathway during inflammatory processes^22^.

A hallmark of CCC is an intense inflammatory process that is clinically persistent in indeterminate and chronic chagasic patients and is essentially characterized by myocarditis^23^. The prevalence of myocarditis correlates with the severity of clinical heart failure such as vasospasms, decreased blood flow, myocardial ischemia, coronary angiography, impairment of endothelium-dependent coronary vasodilatation, platelet thrombi and increased platelet aggregation^24^,^25^,^26^,^27^,^28^. The micro-vascular ischemia observed during CCC may be partially explained by the active role of P21. Intracellular, trapped parasites may constantly secrete P21 that can gain access to the extracellular space and bind to CXCR4^+^ ECs from the heart that would lose their capacity to promote re-vascularization *in situ* contributing to disease progression. This hypothesis is partially supported by several independent studies showing that the persistence of *T. cruzi* is directly associated with CCC pathogenesis. For example, Andrade *et al*.^29^ and Silva *et al*.^30^ reported that an enhanced parasite burden exacerbates the cardiomyopathy course. Moreover, Añes *et al*.^31^ showed that intact amastigotes are able to survive and multiply inside host heart tissue during the chronic phase contributing to disease progression.

**Figure 3:**
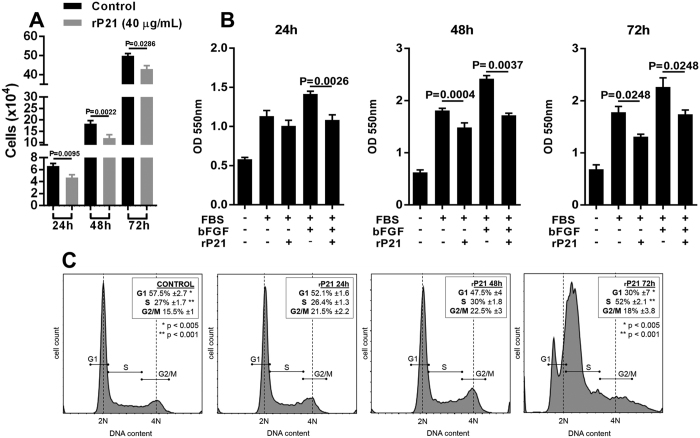
rP21 inhibits EC proliferation and interferes in cell cycle. (**A**) Growth curve of tEnd cells in the presence of rP21 showed a reduction in cell number. (**B**) rP21 inhibited EC proliferation at 24, 48 and 72 h post-treatment and (**C**) promoted a significant decrease in the 2N cell number and increased the cell number in S phase at 72 h post-treatment. Data are expressed as mean ± standard deviation. Significant differences were determined using one-way ANOVA and Tukey’s multiple comparisons test (**A**–**C**). Differences were considered significant when p < 0.05.

The CXCR4-CXCL12 axis is widely known to promote EC multiplication^32^,^33^,^34^,^35^. In this context, we proposed to assess the influence of rP21 on EC proliferation. EC cell growth curve showed a decrease in all time points of rP21 treatment (Fig. 3A). Moreover, MTT assay demonstrated that while the combination of fetal bovine serum (FBS) and basic fibroblastic growth factor (bFGF) saturated EC proliferation capacity, rP21 inhibited EC proliferation at 24, 48 and 72 h post-treatment (Fig. 3B). This anti-proliferative activity of rP21 was confirmed by cell cycle analysis where we observed a significant decrease in the 2N cell number and an increase in the number of cell in S phase up to 72 h (Fig. 3C), raising the possibility that the anti-proliferative activity of rP21 can be related to replicative stress. Taken together, these results suggest that rP21 antagonizes CXCL12 activity. The subsequent mechanisms by which rP21 inhibits cell proliferation will be thoroughly evaluated in future studies from our research group.

### rP21 induces actin cytoskeleton polymerization and modulates the expression of actin-related genes in endothelial cells

Actin is a major cytoskeletal component of ECs and is a well-studied cytoskeletal element that controls angiogenesis^36^. The actin cytoskeleton orchestrates various steps in both physiological and pathological angiogenesis which require continuous cytoskeletal-dependent remodeling^37^,^38^. Thus, the actin network and its associated proteins integrate with and respond to key signaling pathways known to regulate angiogenesis^39^.

**Figure 4:**
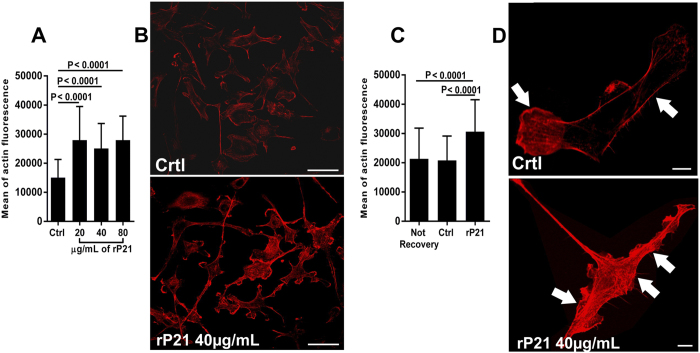
rP21 induces actin cytoskeleton polymerization in ECs. (**A**) Different concentrations of rP21 significantly increased F-actin levels in tEnd cells compared to the untreated group and (**B**) representative images are shown. Bars: 50 μm. (**C**) rP21 treatment promoted faster recovery of the actin filaments compared to the control groups, confirming that rP21 promotes actin polymerization. (**D**) Representative images highlighting the different actin cytoskeleton morphologies where rP21-treated tEnd cells exhibited thick actin fibers and sites of lamellipodial membrane protrusions enriched in F-actin compared to the control group (white arrows). Bars: 10 μm. The data are expressed as the means ± standard deviations of experiments performed in triplicate. Significant differences were determined using one-way ANOVA with Tukey’s multiple comparisons test (**A**,**B**). Differences were considered significant when p < 0.05.

To investigate the influence of rP21 on the actin cytoskeleton of ECs, we treated tEnd cells with different concentrations of rP21 and observed that rP21 significantly increased F-actin levels in ECs compared to the controls (not treated) using fluorescence microscopy (Fig. 4A,B). The effect of rP21 on the F-actin level may be due to an increase in the rate of actin polymerization, a decrease in actin depolymerization or both. To assess which of these processes are regulated by rP21, we performed an F-actin recovery assay using cytochalasin-D (CytD). tEnd cells were treated with CytD (1.5 μM) for 40 min to inhibit actin subunit assembly followed by a recovery period in the presence or absence of rP21. We observed that rP21 increased the recovery rate of the actin filaments compared to the control groups. Thus, these results showed that rP21 has a remarkable capacity to promote actin polymerization in accordance with our previous studies^19^ (Fig. 4C).

The rP21-treated tEnd cells had markedly different actin cytoskeleton morphology compared to the control cells. ECs exhibited long, thick actin stress fibers that were aligned along cell longitudinal axis, and the plasma membrane was aligned with the bright phalloidin staining. In contrast, control cells had wispy, short actin filament bundles, and their plasma membrane was weakly stained with phalloidin. Moreover, rP21-treated tEnd cells exhibited sites of lamellipodial membrane protrusions enriched in F-actin compared to the control group (Fig. 4D - white arrows).

**Figure 5:**
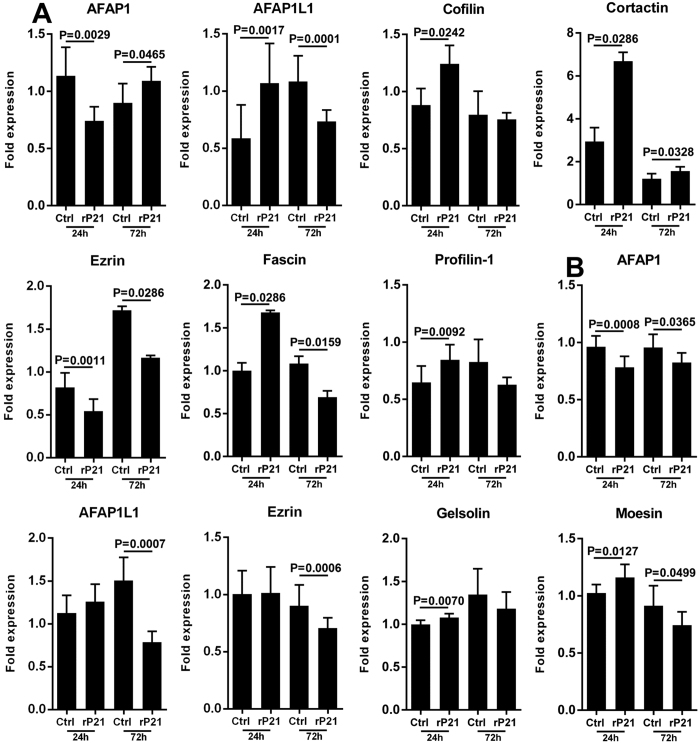
rP21 modulates the expression of actin-related genes in ECs. (**A**) Differential expression of actin-related mRNAs in tEnd cells was analyzed by RT-qPCR after 24 and 72 h of rP21 stimulation in 2-D and (**B**) 3-D cell cultures. Data are expressed as means ± standard deviations of experiments performed in triplicate. Significant differences were determined using Student’s t-test (two-sided) and Mann-Whitney test. Differences were considered significant when p < 0.05.

To gain insights into the direct effect of rP21 on actin cytoskeleton dynamics, tEnd cells were seeded into 24-well plates and incubated with rP21 or culture medium (control group) for 24 and 72 h and actin cytoskeleton-related gene expression was analyzed. rP21 modulated the expression levels of 7 important genes involved in a number of different biological effects such as cell motility, migration, morphogenesis, cell-cell interactions and cell-ECM component interactions. rP21 up-regulated the expression of actin filament-associated protein 1-like 1 (AFAP1L1), cofilin, cortactin, fascin, profilin-1 and down-regulated the expression of ezrin and actin filament-associated protein 110 kilodaltons (kDa) in size (AFAP-110/AFAP1) at 24 h. Interestingly, at 72 h of rP21 treatment we observed a turnover in gene expression with the exception of cofilin, profilin-1, ezrin and cortactin (Fig. 5A). Moreover, rP21 did not alter the expression of gelsolin, moesin and actin-related proteins 2 and 3 (ARP2/3) complex genes (data not shown).

In this context, we examined whether changes in the mechanical properties of the substrate could promote the same effects on the expression of genes related to the actin cytoskeleton in rP21-treated ECs. Thus, tEnd cells were plated in 24-well plates that had been previously coated with a thin layer of ECM. After 24 and 72 h of treatment with or without rP21, total RNA was extracted and analyzed by real-time PCR (RT-qPCR). We observed different gene expression profiles between two-dimensional (2-D) and three-dimensional (3-D) cell cultures. At 24 h of treatment in 3-D cultures, rP21 promoted a slight down-regulation of AFAP1 and ARP2 expression and up-regulated gelsolin and moesin expression. Curiously, after 72 h rP21 only promoted down-regulated AFAP1, AFAP1L1, ezrin and moesin expression (Fig. 5B). Cofilin, cortactin, fascin, profilin-1 and ARP3 gene expression levels were not modulated by rP21 in the 3-D culture model (data not shown).

Dynamic regulation of the actin cytoskeleton plays a central role in a variety of cellular events and involves a number of actin-binding proteins (ABPs) including capping, branching, severing, sequestering, and cross-linking proteins^40^. Cofilin is a small ubiquitous protein that is able to bind both monomeric (G) and filamentous (F) actin. The F-actin-severing protein cofilin is a key player that regulates the dynamics of actin polymerization and depolymerization in migrating cells^41^,^42^,^43^,^44^,^45^. Several studies have shown that cofilin is required to determine the direction of cell migration through actin polymerization-based protrusions^42^,^46^,^47^. Cortactin, another ABP, is located in the cytoplasm of cells and is associated with the polymerization and rearrangement of actin cytoskeleton, mainly at the periphery of the cellular cortex^48^,^49^. This protein actively triggers lamellipodia, protrusions of the plasma membrane that are characterized by high F-actin levels. Similar to cofilin, cortactin is also involved in cell motility^50^. Fascin is an actin cross-linking protein located in the actin-rich protrusions of ECs and is involved in the regulation of cytoskeletal scaffolds during cell adhesion and migration^51^.

AFAP1 and AFAP1L1 are members of the AFAP family of proteins; as adaptor proteins (non-enzymatic proteins) associated with the assembly and disassembly of the actin cytoskeleton^52^,^53^, these proteins affect cell adhesion, invasion and motility^54^. Snyder *et al*.^55^ showed that AFAP1 and AFAP1L1 overexpression promoted lamellipodia formation in the absence of extracellular signals. Moreover, these results indicate that AFAP1L1, like AFAP1, is associated with actin-stress fiber formation. In their report, the authors showed that AFAP1L1 but not AFAP1 binds cortactin to form a complex. Thus, these proteins may have the capacity to interact with different proteins involved in actin cytoskeleton dynamics. Ezrin, radixin and moesin, which form the ERM protein family, act as linkers between the plasma membrane and the cortical actin filaments and are involved in many physiological functions including regulation of the actin cytoskeleton, control of cell shape, adhesion, lamellipodia formation, motility and modulation of signal transduction pathways. Ezrin plays a key role in the actin-based cellular functions required for cell locomotion that are important in angiogenesis^56^,^57^,^58^.

Literature shows that different ABPs play critical roles in regulating the complex series of signaling events in ECs to shape the changes that occur during migration and angiogenesis. In this context, the results of our 2-D cell cultures showed that rP21 is able to up-regulate the expression of genes that are required for actin polymerization, stress fiber formation and migration such as cofilin, cortactin, AFAP1, AFAP1L1, and fascin. However, when gene expression levels from 3-D cell cultures were determined essentially the very genes that were overexpressed in 2-D cultures did not show altered expression or were down-regulated by the rP21 treatment, with the exception of gelsolin and moesin at 24 h, which were slightly up-regulated. Interestingly, ezrin (ERM protein family) expression was significantly down-regulated in both cell culture systems. The negative modulation of ezrin may be directly related to the inhibition of blood vessel formation. This hypothesis is corroborated by Zhao *et al*.^59^ that showed that silencing of the ezrin gene in human umbilical vein endothelial cells (HUVECs) *in vitro* suppressed migration and angiogenesis.

It is known that the cytoskeletal organization within ECs in 2-D cell cultures is distinct from that in 3-D cultures^36^,^59^,^60^,^61^,^62^,^63^. Growing evidence from 2-D model systems has suggested that mechanical substrates (i.e., rigid glass or polystyrene substrates) promote changes in migration, proliferation, differentiation and cell shape. However, it remains unclear whether these physiological processes may be generalized to 3-D cultures or *in vivo* systems^59^,^60^,^64^. It has clearly been shown that ECs extend sprouts into an intact 3-D ECM whereas ECs form a thin monolayer upon a mechanical substrate. Therefore, ECs behave differently when grown in 3-D cultures compared to 2-D cultures^36^. In this sense, the differences in gene expression profiles observed in our data may be due changes in the matrix composition that can directly or indirectly influence myosin-driven, actin-mediated contractility, cell motility, cell-cell interactions and/or intracellular signaling pathways^61^.

### rP21 modulates expression of angiogenesis-associated genes

**Figure 6:**
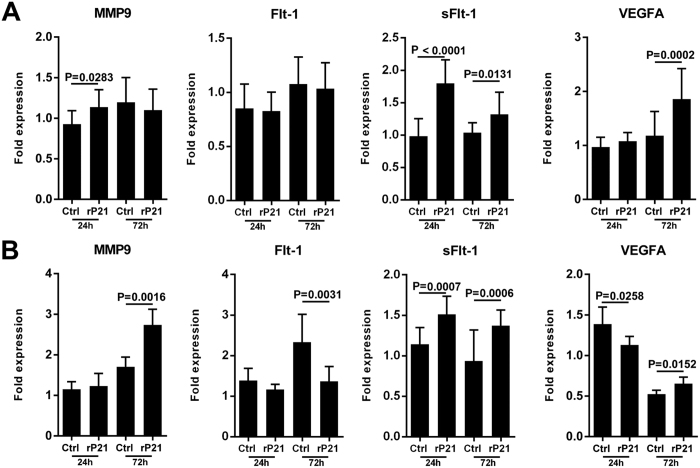
rP21 modulates the expression of angiogenesis-associated genes. (**A**) Gene expression profiles of pro- and anti-angiogenic molecules after 24 and 72 h of rP21 stimulation in 2-D and (**B**) 3-D cell cultures. The data are expressed as the means ± standard deviations of experiments performed in triplicate. Significant differences were determined using Student’s t-test (two-sided) and Mann-Whitney test. Differences were considered significant when p < 0.05.

To assess the effect of rP21 on gene expression profiles of pro- and anti-angiogenic molecules, tEnd cells were plated in 24-well plates that had been previously coated with a thin layer of extracellular matrix (3-D system) or not (2-D system) and incubated with rP21 or culture medium (control group) for 24 and 72 h. After 24 h of treatment in 2-D culture, rP21 up-regulated the expression of matrix metalloproteinase 9 (MMP9) and soluble fms-like tyrosine kinase 1 (sFlt-1). However, rP21 did not alter vascular endothelial growth factor receptor-1/fms-like tyrosine kinase (VEGFR1/Flt1) and vascular endothelial growth factor A (VEGFA) expression. At 72 h, rP21 continued to up-regulate sFlt-1 expression. Interestingly, rP21 promoted a positive modulation of VEGFA expression and did not influence MMP9 and Flt-1 expression at 72 h (Fig. 6A). In 3-D cell cultures, rP21 increased the expression levels of sFlt-1 at 24 h and MMP9, sFlt-1, and VEGFA at 72 h and decreased the expression levels of VEGFA at 24 h and Flt-1 at 72 h (Fig. 6B).

Angiogenesis is known to be controlled by the balance between various pro- and anti-angiogenic signals. MMP9 is an enzyme expressed in many cell types, including ECs, and has the capacity to degrade many molecules of the ECM. Several studies have shown that this metalloproteinase is implicated in the pro-angiogenic process in various ways^65^,^66^,^67^,^68^. However, the role of MMP9 during angiogenesis remains uncertain^69^.

VEGFA belongs to the mammalian platelet-derived growth factor (PDGF) family and acts as a potent, multifunctional cytokine that induces colony formation by recruiting mature subsets of cells. It also plays an active role in the regulation of the physiological and pathological growth of blood vessels formed during angiogenic and vasculogenic processes^70^. VEGFA binds to VEGFR1 (Flt-1) with an affinity that is approximately 10-fold higher than that of Flk-1/KDR (VEGFR2)^71^. VEGFR1 and VEGFR2 are members of the VEGFR tyrosine kinase (TK) family and are highly expressed in ECs. Flt-1 is alternatively spliced to produce both a membrane-localized and a soluble form (sFlt-1) that are secreted by ECs. Shibuya and Claesson-Welsh^71^ showed that VEGFR1-null mice exhibited overgrowth and dysmorphogenesis of blood vessels which suggests that Flt-1 is a negative regulator of angiogenesis during embryonic development. Takeda *et al*.^72^ also revealed the negative regulatory role of Flt-1, as knockout mice exhibited embryonic lethality due to the overgrowth of ECs and blood vessel dysfunction. In addition, Amano *et al*.^73^ showed that a knockout mouse lacking the Flt-1 intracellular TK domain exhibited impaired angiogenesis after hind limb ischemia indicating that Flt-1 signaling is essential for ischemia-related re-vascularization. Furthermore, Murdoch *et al*.^74^ showed that the induction of sFlt-1 expression is relevant to the recovery of suppressed blood flow following hind limb ischemia. It is known that sFlt-1 retains high affinity for VEGFA^75^ and can trap VEGFA to prevent it from binding to VEGFRs. These results suggest that soluble Flt-1 modulates the amount of VEGFA^76^ and inhibits VEGFA activity without affecting the intracellular signaling pathways of VEGFRs^73^. Thus, Flt-1 negatively regulates angiogenesis through its extracellular domain and positively regulates angiogenesis through its tyrosine kinase domain.

According to the results of the 3-D cellular culture system, we suggest that the anti-angiogenic activity of rP21 at 24 h is related to the up-regulation of sFlt-1 expression and the negative modulation of VEGFA. However, at 72 h we observed a decrease in sFlt-1 expression and increase in VEGFA and MMP9 expression in rP21-treated ECs. Interestingly, at this time point we observed that rP21 strongly down-regulated Flt-1 expression that may be related to the negative regulation of angiogenesis in agreement with the studies mentioned above. In this sense, the initial inhibition of angiogenesis by rP21 at 24 h could explain the increased sFlt-1 levels and the continued inhibition of angiogenesis at 72 h is likely related to the down-regulation of Flt-1. In the 2-D system, rP21 positively regulated the pro- and anti-angiogenic signals. However, it is known that the cells have different behaviors that depend on the conditions of the cell culture model system.

**Figure 7:**
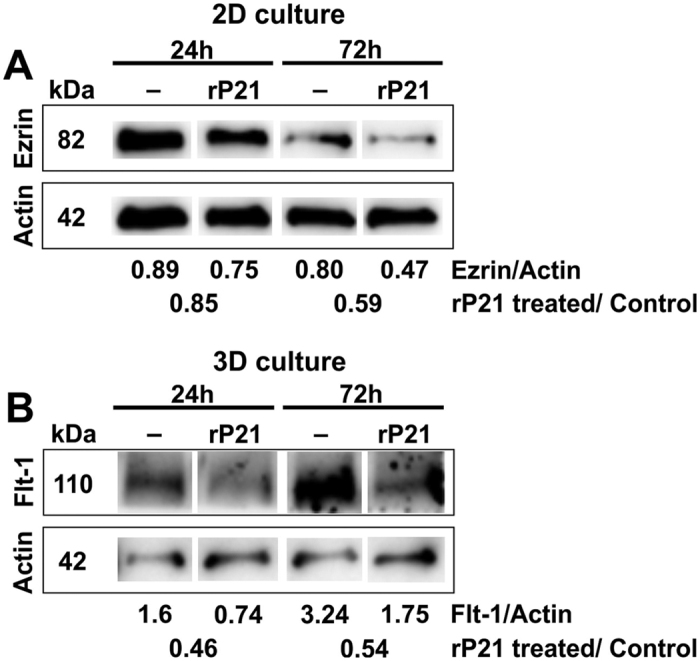
rP21 treatment interferes in the expression of actin cytoskeleton and angiogenesis-related proteins. (**A**) After 24 and 72 h, rP21 promoted a robust reduction in ezrin expression (2-D system) and (**B**) Flt-1/VEGFR-1 (3-D system). Actin was used as a loading control. Cropped blots are displayed.

In order to support our gene expression results, we performed immunoblotting analysis of ezrin and Flt-1 protein expression in 2-D or 3-D cultured cells treated or not with rP21. Our results showed decreased expression of ezrin in 2-D cultured cells treated with rP21 (Fig. 7A). Also, we observed a significant decrease in Flt-1 protein content from 3-D cells previously treated with rP21 (Fig. 7B).

### Potential P21-mediated mechanism during CCC pathogenesis

**Figure 8:**
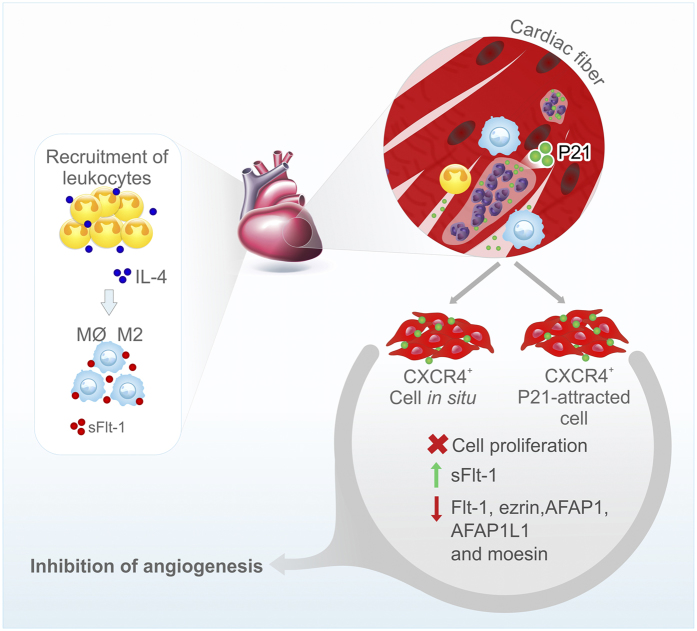
Potential P21-mediated mechanism during CCC pathogenesis. P21 is continuously secreted to the extracellular space by intracellular amastigotes located in cardiac fibers and induces leukocyte recruitment to the site of inflammation and up-regulates IL-4 expression. IL-4 induces macrophages to acquire alternative (M2) activation that is characterized by increased production of sFlt-1 (anti-angiogenic molecule). Moreover, P21-sensitized CXCR4^+^ ECs would lose their capacity to promote *in situ* re-vascularization through a cascade of intracellular events such as inhibition of EC proliferation, sFlt-1 overexpression, and down-regulation of Flt-1, ezrin, AFAP1, AFAP1L1 and moesin. PCBT have drawn the figure.

Combining our findings and the current literature, we hypothesize that an anti-angiogenic mechanism is triggered by P21 during CCC onset and progression. We propose that P21 is continuously secreted to the extracellular space by intracellular amastigotes located in cardiac fibers that induces leukocyte recruitment to the site of inflammation and up-regulates IL-4 expression^15^. It is known that IL-4 induces macrophages to acquire alternative (M2) activation that is characterized by increased production of sFlt-1 (anti-angiogenic molecule)^77^. Moreover, we believe that P21 has the capacity to bind to CXCR4^+^ ECs from the heart. P21-sensitized ECs would lose their capacity to promote re-vascularization *in situ* through a cascade of intracellular events such as inhibition of EC proliferation, sFlt-1 overexpression, and down-regulation of Flt-1, ezrin, AFAP1, AFAP1L1 and moesin, that could partially explain the functional and structural micro-vascular abnormalities observed in CCC^13^ (Fig. 8).

### Experimental procedures

#### Cell line and culture

A murine EC line derived from a thymus hemangioma (tEnd) was used as established by Bussolino *et al*.^78^ and Achê *et al*.^79^ with some modifications. Cells were cultivated in Dulbecco’s Modified Eagle Medium (DMEM) supplemented with 10% FBS, 2 mM l-glutamine, 2 mM sodium pyruvate, 1 mM non-essential amino acids, 100 U/mL penicillin and 100 μg/mL streptomycin and incubated at 37 °C in a humidified atmosphere containing 5% CO_2_.

#### rP21 purification

rP21 (GenBank: EU004210.1) was purified as previously described by Dos Santos *et al*.^80^.

#### Matrigel tube formation assay

The influence of rP21 on EC tube formation was evaluated as previously described^81^, with modifications. tEnd cells (5 × 10^5 ^cells/well) were pre-incubated with rP21 (40 μg/mL), AMD3100-CXCR4 inhibitor (30 μM) or culture medium for 30 minutes at 37 °C. To evaluate whether anti-angiogenic activity of rP21 occurs by direct interaction with EC, tEnd cells pre-incubated with rP21 (40 μg/mL) were also washed out with PBS and centrifuged (5 min, 1500 RPM). After treatment, cells were seeded at 24-well plates that had previously been coated with 50 μL of 5.25 mg/mL Matrigel (BD Bioscience) and incubated with medium supplemented with bFGF (30 ng/mL). After 24, 48 and 72 h of incubation at 37 °C and 5% CO_2_, images were acquired at 20x magnification using a brightfield microscope and the number of vessels counted^15^.

#### Degradation of ECM components

Degradation of ECM components was assayed as previously described^18^,^82^. To evaluate the fibrinogenolytic activity 50 μL of bovine fibrinogen sample (1.5 mg/mL in PBS, pH 7.8) was incubated with different amounts of rP21 (5, 10, 20 and 40 μg) for 1 h at 37 °C. The reaction was stopped with 25 μL stop solution (0.06 M Tris-HCl, pH 6.8, containing 10% (v/v) glycerol, 10% (v/v) β-mercaptoethanol, 2% (w/v) SDS and 0.05% (w/v) bromophenol blue). Samples were then heated at 100 °C for 5 minutes and analyzed by 12.5% (w/v) SDS-PAGE.

Matrigel (5.25 mg/mL) or a fibronectin solution (1 mg/mL in 50 mM Tris-HCl, pH 7.4) was incubated with 40 μg of rP21 at 37 °C for 1 h. The reaction was stopped by adding stop solution, samples were heated, and the substrate digestion was analyzed by 7% SDS-PAGE (w/v), as mentioned above.

#### Protein binding and internalization assay

Direct interaction of rP21 with ECs was assessed as previously described^83^,^84^. tEnd cells were seeded at a density of 2.5 × 10^4 ^cells/coverslip in 24-well micro-plates and were then incubated with rP21 for 24 h. After washing, cells were fixed with 4% paraformaldehyde for 1 h at room temperature and washed three times with PBS. Cells were incubated overnight with an anti-rP21 primary polyclonal rabbit antibody (diluted 1:200 in PGN-0.01% saponin solution). Then, coverslips were washed and incubated with Alexa Fluor 488-conjugated anti-rabbit IgG (1:200 in PGN + saponin), tetramethyl rhodamine isothiocyanate (TRITC)-conjugated phalloidin (diluted 1:500 in PGN + saponin) and 4,6′-diamidino-2-phenylindole dilactate (DAPI, Invitrogen, USA) (diluted 1:500 in PGN + saponin) for 1 h. Coverslips were mounted on glass slides and samples were analyzed by confocal fluorescence microscopy (Zeiss, LSM 510 Meta, Germany) using an inverted microscope (Zeiss Axiovert 200 M).

#### CXCR4 receptor expression in endothelial cells

To examine CXCR4 receptor expression tEnd cells (1 × 10^6^ cells) were fixed with 4% formaldehyde for 1 h washed with PBS and then stained with an anti-CXCR4 HU CD184 BV 421 12G5 50 TST antibody diluted in PBS. Samples were analyzed in a FACSCantoII (BD) and the results were obtained using FlowJo software (version 7.6.3).

#### Proliferation assays

tEnd cells were seeded at a density of 1.0 × 10^4^ cells in 24-well micro-plates. After adhesion, cells were incubated with culture medium or rP21 (40 μg/mL) for 24, 48 and 72 h at 37 °C. Following treatment, cells were harvested and counted in Neubauer chamber.

Additionally, the proliferation of the rP21-treated tEnd cells also was evaluated using MTT (3-(4,5-dimethylthiazol-2-yl)-2,5-diphenyl tetrazolium bromide) assay^83^. Briefly, cells were seeded at a density of 1 × 10^4 ^cells/well in 96-well micro-plates. After adhesion, cells were grown in medium supplemented with FBS and bFGF in the presence or absence of 40 μg/mL rP21 for 24, 48 and 72 h at 37 °C and 5% CO_2_. As a negative control, cells were grown in growth factor- and FBS-free medium. After treatment, cells were incubated with 5 mg/mL MTT for 3 h at 37 °C. Formazan crystals resulting from MTT reduction were dissolved by the addition of 100 μL of phosphate-buffered saline (PBS) containing 10% SDS and 0.01 M HCl (18 h, 37 °C and 5% CO_2_). Absorbance (550 nm) was measured on a multi-well scanning spectrophotometer (Thermo Scientific).

#### Cell cycle analysis

Cell cycle was analyzed by quantifying the DNA content as previously described^85^,^86^. Briefly, tEnd cells were plated at a density of 0.25 × 10^5 ^cells/well in 24-well micro-plates and after adhesion cells were treated with rP21 (40 μg/mL) or culture medium (control group) for 24, 48 and 72 h at 37 °C and 5% CO_2_. Then, cells were harvested and fixed in 70% ethanol overnight at 4 °C. To ensure that only the DNA was stained, cells were incubated with RNase A (100 μg/mL) and propidium iodide (PI) (10 μg/mL) for 45 minutes at 37 °C. Cell cycle was analyzed in a FACSCantoII (BD) and the data were obtained using FlowJo software (version 7.6.3).

#### F-actin staining and actin recovery assay

F-actin accumulation and distribution were determined as described^87^. tEnd cells were seeded at a density of 2.5 × 10^4 ^cells/coverslip in 24-well micro-plates. After adhesion, cells were treated with different rP21 concentrations (20, 40, and 80 μg/mL) or culture medium (control group) for 90 min at 37 °C and 5% CO_2_. Then, cells were fixed with 4% formaldehyde for 1 h at room temperature and coverslips washed three times with PBS. Cells were incubated with TRITC-conjugated phalloidin (diluted 1:500 in PGN-0.01% saponin solution) and DAPI (diluted 1:500 in PGN + saponin) for 1 h to label F-actin and nuclei, respectively.

F-actin recovery experiment was performed as previously described^87^,^88^. tEnd cells were seeded at a density of 2 × 10^4 ^cells/coverslip in 24-well micro-plates. After adhesion, cells were treated with 1.5 μM cytochalasin-D (a pharmacological inhibitor of actin subunit assembly at the filament barbed ends) for 40 minutes to induce actin disassembly, followed by a 90 min recovery period in the presence of rP21 (40 μg/mL) or culture medium (control group), during which F-actin would be re-assembled. Then, cells were submitted to the same labeling conditions described above. In both experiments, the coverslips were mounted on glass slides and images captured with a 63× oil immersion objective using an inverted fluorescence microscope (Zeiss Axiovert 200 M). Digital images were analyzed using confocal fluorescence microscopy software (Zeiss, LSM 510 Meta, Germany) and the mean F-actin fluorescence was determined by setting a high threshold in ImageJ software (National Institutes of Health, USA)^88^.

#### Gene expression

For the real-time PCR experiments, tEnd cells (1 × 10^4^ cells/well) were seeded in 24-well micro-plates that had previously been coated with a thin ECM (3-D cell culture system) or not (2-D cell culture system). Cells were then incubated with rP21 (40 μg/mL) or culture medium (control group) for 24 and 72 h. Total RNA was extracted using a RiboZol™ Plus RNA Purification Kit (Amaresco) and a High Capacity cDNA Reverse Transcription Kit (Applied Biosystems) was used for reverse transcription according to the manufacturers’ instructions. Quantitative RT-PCR was performed using an ABI 7300 system (Applied Biosystems) and SDS v1.4.1 Software (Applied Biosystems) was used to analyze the received data. Reaction consisted of 5 μL of SYBR^®^ Green PCR Master Mix (2X) (Applied Biosystems), 10 μM forward and reverse primers (0.5 μL F + 0.5 μL R), 4 μL of nuclease-free water, and 2 μL of cDNA (125 ng/μL) in a total volume of 12 μL per reaction/sample. The following thermal cycling protocol was used, as recommended by the manufacturer: 95 °C for 10 min, 95 °C for 15 seconds and 60 °C for 1 min for 40 cycles. The cycles were followed by a melting curve analysis at 95 °C for 15 seconds and 60 °C for 1 min.

Primer sequences were designed using sequence alignments obtained from GenBank (NIH/NCBI) based on the published RNA sequences (Supplementary Table 1). Data were normalized using Beta-2 microglobulin (β2M) as a housekeeping gene and then analyzed via the comparative threshold cycle (C_T_) method to calculate fold changes in expression in rP21-treated groups compared to the control group where ∆C_T_ = the C_T_ of gene of interest minus the C_T_ of B2m and ∆∆C_T_ = ∆C_T_ of the rP21-treated groups minus the ∆C_T_ of culture medium-treated groups. The fold changes in gene expression for the rP21-treated groups were then calculated as 2^∆∆CT^. All real-time experiments were performed with biological and technical triplicates.

#### Immunoblotting analysis

To determine protein expression, tEnd cells were seeded in 24-well micro-plates that had previously been coated with a thin ECM (3-D cell culture system) or not (2-D cell culture system). Cells were then incubated with rP21 (40 μg/mL) or culture medium (control group) for 24 and 72 h. Following PBS washes, cells were harvested in RIPA (Radioimmunoprecipitation) Lysis Buffer (pH 7.4) (Santa Cruz Biotechnology) containing 200 mM PMSF (phenylmethane sulfonyl fluoride), protease inhibitor cocktail and 100 mM sodium orthovanadate. After sodium dodecyl sulfate-polyacrylamide gel electrophoresis (SDS-PAGE) and electrophoretic protein transfer, the membrane was incubated with monoclonal anti-Ezrin (Sigma-Aldrich/E 8897) and VEGFR-1/Flt-1 antibody (R&D Systems/AF471) (gifts from Professor Ricardo José Giordano, Universidade de São Paulo, Brazil) followed by horseradish peroxidase-conjugated secondary antibody (Sigma-Aldrich). Immunoreactive signals were visualized by using enhanced chemiluminescence (Amersham), and densitometry performed using UVIBAND image quantification software. Loading controls of extracts were checked with anti-actin antibody (Sigma-Aldrich).

#### Statistical analysis

Data are expressed as mean ± standard deviation of experiments performed at least three times in triplicate. All data were first checked for normal distribution. Significance differences were determined by one-way ANOVA, Tukey’s multiple comparisons test and Student’s t-test (two-sided) for the parametric data or the Mann-Whitney test for nonparametric data according to the experimental design (GraphPad Prism Software version 6.01). Data were considered statistically significant at p < 0.05.

## Additional Information

**How to cite this article:** Teixeira, S. C. *et al*. Mechanistic Insights into the Anti-angiogenic Activity of *Trypanosoma cruzi* Protein 21 and its Potential Impact on the Onset of Chagasic Cardiomyopathy. *Sci. Rep.* **7**, 44978; doi: 10.1038/srep44978 (2017).

**Publisher's note:** Springer Nature remains neutral with regard to jurisdictional claims in published maps and institutional affiliations.

## Supplementary Material

Supplementary Table 1
