# Supplementary material for: Mechanistic Insights into the Anti-angiogenic Activity of Trypanosoma cruzi Protein 21 and its Potential Impact on the Onset of Chagasic Cardiomyopathy
Source: Sci Rep. 2017 Mar 21;7:44978. doi: 10.1038/srep44978 (PMC5359584; doi:10.1038/srep44978)
Supplement: Supplementary Table 1 [file srep44978-s1.doc]

**Mechanistic Insights into the Anti-angiogenic Activity of *Trypanosoma cruzi* Protein 21 and its Potential Impact on the Onset of Chagasic Cardiomyopathy**

Samuel Cota Teixeira^1^, Daiana Silva Lopes^2^, Sarah Natalie Cirilo Gimenes^2^, Thaise Lara Teixeira^1^, Marcelo Santos da Silva^3^, Rebecca Tavares e Silva Brígido^4^, Felipe Andrés Cordero da Luz^5^, Aline Alves da Silva^1^, Makswell Almeida Silva^2^, Pilar Veras Florentino^6^, Paula Cristina Brígido Tavares^1^, Marlus Alves dos Santos^1^, Veridiana de Melo Rodrigues Ávila^2^, Marcelo José Barbosa Silva^5^, Maria Carolina Elias^3^, Renato Arruda Mortara^6^, and Claudio Vieira da Silva^1,*^

**Supplementary Table 1 - Specific mouse primer pairs.**

| **Gene ID** |  | **Sequence** | **Reference sequence** |
| --- | --- | --- | --- |
| **AFAP1** | **FW** | CCATCGTAGGCTACAAGGAC | NM_011072.4 |
|  | **RV** | CAACCTCAGCTGGCGTAATG |  |
| **AFAP1L1** | **FW** | CGACTGGGACGAGACAAATAC | NM_010833.2 |
|  | **RV** | CCCACTACATGGACTCAAACTC |  |
| **ARP2** | **FW** | TCTCACCCACCTCATTCCTT | NM_007984.2 |
|  | **RV** | CCTAGGGCCTTTCAACAGTAAC |  |
| **ARP3** | **FW** | GAGGCGACAGCTACATCATT | NM_001206367.1 |
|  | **RV** | AACCTCATCCTGGGTAGACT |  |
| **Cofilin-1** | **FW** | CTCTCCCTGTCAACTGTGTTTC | NM_178928.4 |
|  | **RV** | TTTCCCATTCCTTGGCCTTC |  |
| **Cortactin** | **FW** | GTGACTCTGTGCTTGTCTGT | NM_007687.5 |
|  | **RV** | CTGCTTCCATGAGTGGTCAA |  |
| **Ezrin** | **FW** | CATCGCAGATGCCCTCATATC | NM_007803.5 |
|  | **RV** | GCAGAGGCTTTCCACATCTT |  |
| **Fascin** | **FW** | CAGGCGCAGGATCAGATAAA | NM_009510.2 |
|  | **RV** | CTCTTCTACCTCGTCCTCCTT |  |
| **Gelsolin** | **FW** | GCACTGGGTTTGTGAAGTGT | NM_146243.2 |
|  | **RV** | CCCACTTTGGTGGTTGATCTG |  |
| **MMP9** | **FW** | CCATGTTCAGGGACTTTGGA | NM_023735.2 |
|  | **RV** | AGGCTTGGGCTTCAATCTAC |  |
| **Moesin** | **FW** | CCACTTGTGGCTACCTGAAT | NM_027373.2 |
|  | **RV** | CCTGTCCTTGTGGAGAATGAG |  |
| **Profilin-1** | **FW** | GGGAGACAATGGGATGAAAG | NM_001025250.3 |
|  | **RV** | GTCGTGTTTCTGGAAGTGAG |  |
| **sFlt-1** | **FW** | AGAAGACTCGGGCACCTATG | D88690.1 |
|  | **RV** | AGGTTTTGAAGCAGGTGTGG |  |
| **VEGFA** | **FW** | CCATCACTCTGAACCTTGTC | NM_010228.3 |
|  | **RV** | CTAACGAGAACTTCTGTCTTCC |  |
| **VEGFR1/Flt-1** | **FW** | CATAGAGGAAGCCCATTACAG | NM_013599.3 |
|  | **RV** | ACATTTGACGTCCAGAGAAG |  |
| **βeta-2 microglobulin** | **FW** | CATAGAGGAAGCCCATTACAG | NM_009735.3 |
|  | **RV** | ACATTTGACGTCCAGAGAAG |  |
